# Comparative Study of Synthesis Methods to Prepare New Functionalized Adsorbent Materials Based on MNPs–GO Coupling

**DOI:** 10.3390/nano10020304

**Published:** 2020-02-11

**Authors:** Pablo Montoro-Leal, Juan Carlos García-Mesa, María del Mar López Guerrero, Elisa Vereda Alonso

**Affiliations:** Department of Analytical Chemistry, Faculty of Sciences, University of Málaga, 29071 Málaga, Spain; montorolealpablo@uma.es (P.M.-L.); jcgm95@uma.es (J.C.G.-M.)

**Keywords:** solid-phase extraction, magnetic nanoparticles, graphene oxide, synthesis: material characterization

## Abstract

In this work, the synthesis of new adsorbent nanomaterials based on the coupling of magnetic nanoparticles and graphene oxide (MNPs–GO) was addressed. Separately, MNPs and GO have adsorbent properties of great interest, but their use involves certain difficulties. The coupling seeks compensation for their disadvantages, while maintaining their excellent properties. Three different routes to synthesize coupled MNPs–GO were studied and are compared in this work. The three synthesized materials were functionalized with chelating groups: [1,5-bis (di-2-pyridyl) methylene] thiocarbonohydrazide (DPTH) and [1,5-bis(2-pyridyl)3-sulfophenylmethylene] thiocarbonohydrazide (PSTH). The new adsorbent nanomaterials were characterized adequately. Moreover, their capacities of adsorption toward heavy and noble metals were determined, in order to apply them as extractants in magnetic solid-phase extraction to preconcentrate metals in environmental samples. The results showed that one of the routes provided nanomaterials with better adsorbent characteristics and higher yields of functionalization.

## 1. Introduction

Heavy metals are a group of chemical agents widely studied in pollution [1]. Heavy metals arise from rocks weathering and oil and carbon combustion [2]. This group is found in nature in trace amounts (concentration lower than 100 mg/L). The heavy metals hazard lies in the difficulty to degrade them, producing bioaccumulation. Platinum-group elements (PGEs: Pt, Pd, Ir, Ru, Os and Rh) and other noble metals, such as Au and Ag, are known for being nonreactive elements. Their presence arises from their wide usage in jewellery, industry and catalysis, for example, in engines of vehicles. Nowadays, these catalysts are a new source of pollution [3]. In ecosystems, these metals can become toxic substances in the presence of certain substances, such as chlorides. Moreover, PGEs are transported to long distances in particles form, appearing in a huge variety of locations (Alpine snow, urban environments, etc.) [4,5]. The development of effective methods for elimination and determination of heavy metals [6] and noble metals is necessary. Moreover, many analytical methods use solid-phase extraction (SPE) as sample pretreatment because it offers high enrichment factors, complete recovery, rapid phase separation and possible automation with different detection techniques [7]. The SPE methods use less sample volume and reagents [8] and present three successive basic steps: First, the adsorbent is conditioned with a solvent. Then, the loading solution is introduced through the solid phase, to retain the analytes, and, finally, the analytes are collected with an appropriate eluent solution [9].

The main objective of this work was the optimization of the synthesis of new adsorbents for heavy and noble metals. The synthesis of these materials was based on the coupling of magnetic nanoparticles (MNPs) and graphene oxide (GO), since both materials possess excellent adsorbent properties. GO is a graphite mono-layer with a two-dimensional plane structure, and it has been exploited as a solid-phase material due to its large specific surface area (2.630 m^2^/g) and numerous oxygen-containing functional groups on the basal plane and the sheet edge (epoxy, carboxylic acid, carbonyl and hydroxy groups) [10]. As a result, a hydrophilic character is provided, resulting in high sorption capacity for the retention and preconcentration of heavy metal ions [11,12]. Moreover, these functional groups cause attraction and repulsion electrostatic forces, stabilizing GO suspension [13,14]. GO adsorption is good, but tedious, slow and high sample volumes are necessary. An exact structural model for GO cannot be proposed, because the functional groups and their distribution over the sheet depend on the oxidation grade, the synthesis method and the starting materials [15,16,17].

Iron oxide MNPs (Fe_3_O_4_) have been exploited as solid-phase materials due to their biocompatibility, degradability, physiological and chemical stability, low toxicity and high magnetic response [18,19]. For these reasons, MNPs have been used as magnetic carriers in medicine, to decontaminate wastewater, preconcentrate anion and cation, etc. [20]. The high magnetic response allows a variation of classical SPE denominated magnetic solid-phase extraction (MSPE). However, NPMs are easily oxidizable and may lose magnetism. With the coupling of MNPs and GO, the use of centrifugation and filtration processes to separate MNPs–GO and solution matrix are avoided [21,22,23]. Furthermore, the coupled MNPs–GO seeks compensation for the disadvantages of MNPs, favoring the dispersion over GO layer and avoiding the agglomeration [17], which could decrease the specific surface area. In addition, GO protects MNPs from atmospheric oxygen. Therefore, GO–MNPs tandem could become an excellent magnetic adsorbent material to develop a MSPE pretreatment method [17,24].

Furthermore, a chemical factor needs to be added in order to increase the selectivity of the coupling to metals. Thus, two chelating agents were considered: [1,5-bis (di-2-pyridyl) methylene] thiocarbonohydrazide (DPTH) and [1,5-bis(2-pyridyl)3-sulfophenylmethylene] thiocarbonohydrazide (PSTH). DPTH can act as chelating agent, while PSTH can act both as chelating agent and as ionic exchanger, due to the presence of the sulfonic group in the structure. DPTH and PSTH are generic complexing agents that contain nitrogen and sulfur atoms with lone electron pair, which can coordinate to a single metal center. These reagents have been previously anchored to different adsorbent materials, such as silica gel, mesoporous silica and magnetic nanoparticles (MNPs), by the research group. Moreover, it is well-known that DPTH and PSTH form metallic complexes with Ir, Pd, Pt [5], Sb [25], Hg [26] and others [27].

In this manuscript, a comparative study of the materials obtained by coupling MNPs and GO on three different synthesis ways—two of them described in the bibliography [7,17] and a new one proposed by the group—has been carried out in order to obtain improved adsorbents, showing higher yields of functionalization. In the proposed method, a fraction of silica-coated MNP was immobilized by covalent bonding to the GO layer, while the rest was immobilized on the GO layer by physical interactions, which was called double coupling. The double coupling allowed the double functionalization of the material (the chelating agent was linked through both amine groups of MNPs and –COOH groups of GO). In this paper, for the first time, to the best of our knowledge, the double functionalization through MNPs and GO is described.

## 2. Materials and Methods

### 2.1. Instrumentation

Mass spectra were obtained with a Trace DSQ Mass spectrometer from Thermo Electron Corporation, (Beverly, MA, USA); the sample was introduced by means of a DIP (direct introduction probe). FTIR Spectra were recorded on a Spectrum 100 FTIR spectrometer from Perkin Elmer, Inc., (Concord, ON, Canada), and samples were measured by using potassium bromide pellets, in which the concentrations for the samples were 0.5% (wt/wt) approximately. X-ray photoelectron spectroscopy (XPS) analysis were performed with a Physical Electronics ESCA 5701 instrument (Chanhassen, MN, USA); binding energies (BE) were observed, considering the position of the C 1s peak at 284.8 eV. The residual pressure in the analysis chamber was maintained below 3 × 10^−9^ Torr during data acquisition. The microstructures of the new materials were observed and studied by transmission electron microscopy (TEM) JEOL, JEM-1400 (Peabody, MA, USA) and N_2_ adsorption isotherms, Micromeritics ASAP 2020 V4.02 (Norcross, GA, USA). The composition of the materials (C, N, O, S) was studied by CHNOS elemental analysis from LECO TruSpec Micro CHNSO (St. Joseph, MI, USA), and the content in Fe was measured with a solid-sampling high-resolution continuous-source graphite-furnace atomic-absorption spectrometer (SS-HR CS GFAAS) contrAA 700 from Analytik Jena (Jena, Germany). The adsorption capacity of the materials was determined with an inductively coupled plasma mass spectrometer (ICP-MS), PerkinElmer Nexion 2000 (Concord, ON, Canada) and an inductively coupled plasma optical emission spectrometer (ICP-OES), PerkinElmer Optima 7300 DV (Concord, ON, Canada).

### 2.2. Reagents and Solutions

All the chemicals used were of analytical reagent grade. The 1000 mg/L standard solutions of Cr, Co, Ni, Cu, As, Cd, Hg, Pb, Pd, Ag, Pt and Au were purchased from Merck (Darmstard, Germany). Working solutions were prepared by appropriate dilution of the stock solution with deionized water (18 MΩ cm) prior to use. Graphite powder and sodium chloroacetate were procured from Merck (Darmstard, Germany). For the synthesis of MNPs, ferrous chloride tetrahydrate (FeCl_2_·4H_2_O), ferric chloride hexahydrate (FeCl_3_·6H_2_O), HCl 37% (wt/wt), ammonium hydroxide 30% (wt/wt), methanol and sodium chloride from Merck (Darmstadt, Germany) were used. Ethylenediamine (EDA) and N,N’-dicyclohexylcarbodiimide (DCC) were procured from Aldrich Chemie (Steinheim, Germany).

### 2.3. Preparation of Graphene Oxide

The exfoliation process described by Diagboya et al. was used to prepare GO from natural graphite, being purified by centrifugation cycles [28].

### 2.4. Preparation of MNPs–GO

#### 2.4.1. Method 1

In this method, first, MNPs were synthesized, covered with silica and functionalized; second, the coupling with GO was addressed.

**Figure 1:**
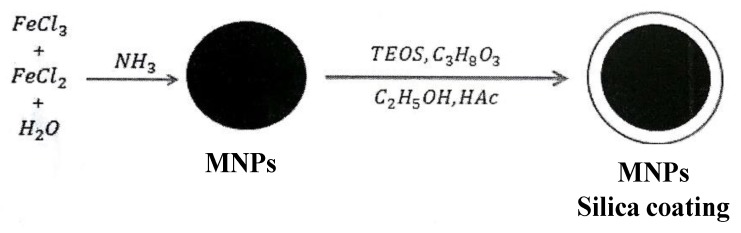
Schematic illustration of preparation of MNPs with silica coating.

*Synthesis of MNPs with silica coating*: FeCl_3_·6H_2_O (11.68 g) and FeCl_2_·4H_2_O (4.30 g) were mixed in 200 mL of deionized water at 80 °C, and then 50 mL of 30% ammonia solution was added quickly, for the coprecipitation of Fe_3_O_4_ nanoparticles in N_2_ atmosphere. The suspension was stirred with reflux for 75 min. The Fe_3_O_4_ solution was cooled to room temperature and separated from the solution with the aid of external permanent magnet and washed with deionized water. The synthesis of MNPs was optimized by González Moreno et al. [29], the prepared MNPs were mixed with 8 mL of TEOS and 60 mL of glycerol in 200 mL of ethanol at 60 °C and stirred for 2 h in N_2_ atmosphere. The resultant suspension was cooled to room temperature and separated from the solution with the aid of external permanent magnet, washed with deionized water and kept in ethanol for further functionalization (Figure 1).

*Coupling MNPs–GO*: This step was made after the functionalization of MNPs, and it was based on the work of Nodeh et al. [17]. The functionalization process was described elsewhere [26,30]. For the coupling, 500 mg of GO and 500 mg of functionalized MNPs were mixed in water and stirred for 10 min. The mix was incubated at 65 °C for 8 h. The coupling Fe_3_O_4_–GO suspension was cooled to room temperature and separated from the solution with the aid of external permanent magnet, and then washed with deionized water and ethanol. Finally, the precipitate was dried at 80 °C for 24 h. The obtained materials were called 1-DPTH and 1-PSTH.

#### 2.4.2. Method 2

GO was made magnetic by slowly adding a 35 mL aqueous solution of mixed FeCl_3_·6H_2_O (1.10 g) and FeCl_2_·4H_2_O (0.43 g) into the GO suspension (300 mg of GO in 200 mL deionized water) at 80 °C, and then 5 mL of 30% ammonia solution was added quickly, for the co-precipitation of Fe_3_O_4_ nanoparticles on GO sheets in N_2_ atmosphere. After being rapidly stirred for 45 min, the resultant Fe_3_O_4_ decorated GO suspension was cooled to room temperature and separated from the solution with the aid of external permanent magnet, washed with deionized water and kept in ethanol for further functionalization [7].

**Figure 2:**
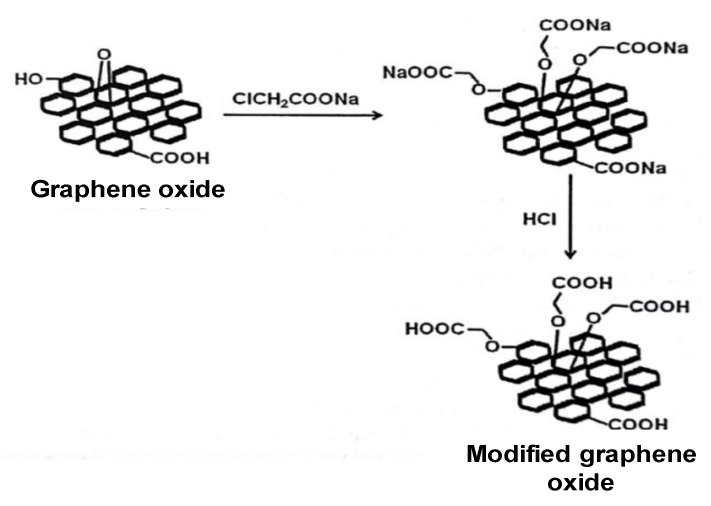
Schematic illustration of preparation of modified GO based on the Du et al. [31].

*MNPs–GO–EDA Coupling*: The prepared material was suspended in 300 mL of deionized water and sonicated for 15 min; then, 3 g of NaOH and 3 g of Cl–CH_2_COONa were added, and the mix was stirred for 2 h [31]. The modified MNPs–GO was separated from the suspension by using an external permanent magnet and washed first with 1% HCl and finally with deionized water. This modification was included to increase the number of –COOH functional groups available over the surface, and, thus, the number of the active sites on the GO surface, by transforming the –OH and epoxide groups into functionalizable –O–CH_2_COOH groups, as is shown in Figure 2. The MNPs–GO–EDA was fabricated “in situ” by coupling of EDA with carboxylic groups of modified MNPs–GO using DCC as a coupling agent. The 1.2 mL of EDA was added into the modified MNPs–GO suspended ethanol solution, along with DCC. The suspension was stirred for 48 h at 50 °C. The –COOH functional groups of the modified MNPs–GO condense with one of the EDA amine groups to form amide bonds, while the second amine group was available to attach an organic group (–CONH–C_2_H_4_–NH_2_). Finally, MNPs–GO–EDA was separated from the suspension, using an external permanent magnet, and washed with deionized water.

#### 2.4.3. Method 3

In this method, double coupling and double functionalization were achieved by anchoring the functional groups with both MNPs and GO.

*MNPs–GO Coupling:* 500 mg of GO was suspended in 50 mL of ethanol, with 500 mg of coated MNPs (synthesized as described in the first step of Method 1) and 0.25 g of DCC in a 100 mL round-bottom flask. The mixture was sonicated for 10 min and kept at reflux at 50 °C for 48 h. In this way, part of the MNPs were covalently anchored to the GO sheet through an amide bond, which is formed by condensation between the acid groups of the GO surface and the amine groups of the MNPs. The rest of MNPs were physically adsorbed on GO surface due to two main factors: performance of the reaction of covalent MNP–GO coupling, which is supposed to be <100%, and the depletion of –COOH functional groups over the GO surface during this synthesis step. Therefore, the MNPs were coupled to the GO through two mechanisms: chemical interactions (covalent binding by condensation with the carboxylic acid groups) and physical interactions (electrostatic and Van der Waals forces) with the GO layer.

*Modification of MNPs–GO:* the solid MNPs–GO from the previous stage was suspended in 50 mL of deionized water. The mixture was sonicated for 15 min, and then 50 mL of NaOH 2.5 M was added. Next, 5 g of sodium acetate chloride (Cl–CH_2_COONa) in 50 mL of deionized water was introduced, and the mixture was maintained for 2 h, in ultrasound, at room temperature. The suspension and the matrix were separated with the aid of an external permanent magnet, and the solid was washed twice, first with 1% HCl and finally with deionized water. This modification increases the number of the active sites on the GO surface, by transformation of –OH and epoxide groups into functionalizable –O–CH_2_COOH groups.

*EDA–MNPs–GO Coupling:* The modified MNPs–GO solid from the previous step was suspended in 50 mL of ethanol, together with 4 mL of EDA and 0.25 g of DCC, in a 100 mL round-bottom flask, and stirred at 50 °C for 48 h. The free acid groups obtained in the previous step were condensed with one of the EDA amine groups to form amide bonds, while the second was available to attach an organic group to functionalize the solid. Thus, there were double-origin amino groups available for functionalization: the amino group of the coated MNPs dispersed on the sheet and the free non-condensed amino group of the EDA.

### 2.5. Functionalization of the Prepared Materials

**Figure 3:**
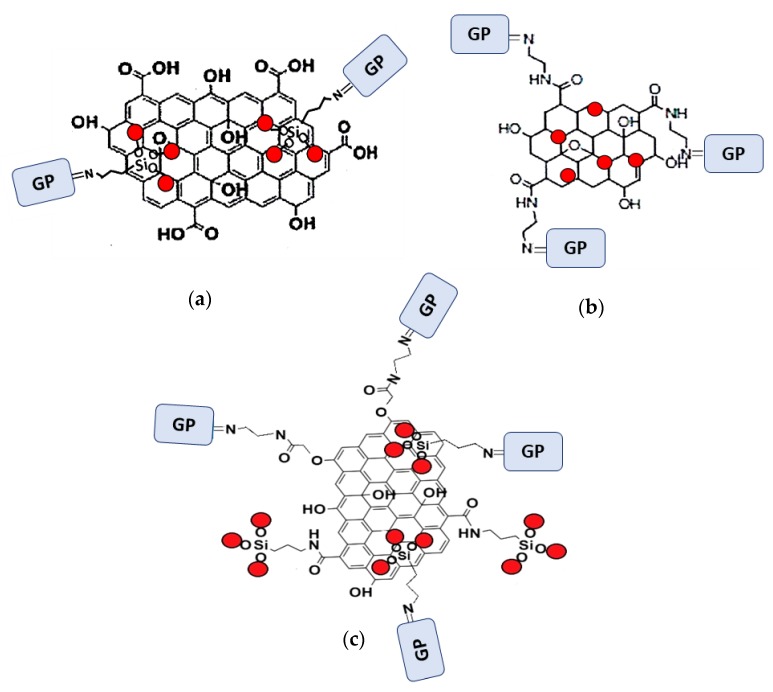
(**a**) Functionalized MNPs–GO structure synthesized by Method 1; (**b**) functionalized MNPs–GO structure synthesized by Method 2 and (**c**) functionalized MNPs–GO structure synthesized by Method 3. GP: Functional Group.

**Figure 4:**
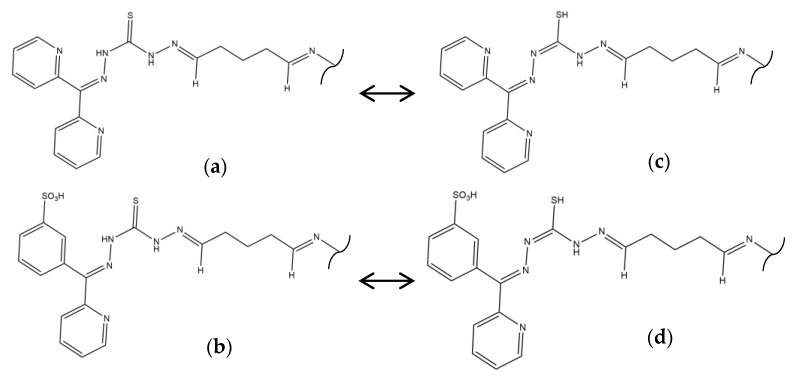
Tautomeric equilibrium of C=S bond (**a**,**c**) DPTH tautomers; (**b**,**d**) PSTH tautomers.

The functionalization processes to attach DPTH or PSTH functional groups were carried out previously by the research group [26,30]. The magnetic materials synthesized following the Methods 1–3 were functionalized and called 1-DPTH, 1-PSTH, 2-DPTH, 2-PSTH, 3-DPTH and 3-PSTH, respectively, where the number is relating to the method. Their schematic structures were presented in Figure 3a–c. The structures of DPTH and PSTH are shown in Figure 4a,b, respectively.

### 2.6. Adsorption of Heavy and Noble Metals from Aqueous Solution

For the study of the capacity of adsorption, samples were prepared by mixing 5 mg of the magnetic material and 50 mL of 50 μg/L of Cd, Co, Cr, Cu, Ni, As, Hg, Pb, Pt, Pd, Ag and Au. The metals were divided in two groups, to avoid the saturation of the resins Cd, Co, Cr, Cu, Ni, As, Hg and Pb (group 1) and Pt, Pd, Ag and Au (group 2). Therefore, there was a total of twelve samples (1-PSTH groups 1 and 2; 2-PSTH groups 1 and 2; 3-PSTH groups 1 and 2; 1-DPTH groups 1 and 2; 2-DPTH groups 1 and 2; 3-DPTH groups 1 and 2). The capacities of adsorption of the materials were studied at acid pH (between 3 and 5). The pH was adjusted by using acetic acid/sodium acetate buffer. The suspensions were stirred for 10 min, and the materials were separated from the suspension by using an external permanent magnet. The decanted solutions were analyzed by ICP-MS. The highest capacity of adsorption that could be obtained was 0.5 mg/g material.

For the materials achieving the maximum capacity of adsorption, a second experiment with higher concentrations of metals was performed. In this case, samples were prepared by mixing 25 mg of the magnetic material and 50 mL of 10 mg/L of Cd, Co, Cr, Cu, Ni, As, Hg, Pb, Pt, Pd, Ag and Au. The metals were divided into three groups, to avoid the saturation of the resins Co, Cr, Cu, Ag and Ni (group 1); As, Hg, Pb and Cd (group 2); and Pt, Pd, and Au (group 3). Therefore, there was a total of six samples (3-PSTH groups 1–3 and 3-DPTH groups 1–3). The capacities of adsorption of the materials were studied at acid pH (between 3 and 5), and the pH was adjusted by using acetic acid/sodium acetate buffer. The suspensions were stirred for 10 min, and the materials were separated from the suspension by using an external permanent magnet. The decanted solutions were analyzed by ICP-OES. The highest capacity of adsorption that could be obtained was 20 mg/g material.

## 3. Results and Discussion

The materials obtained, using the three synthesis methods and the two functional groups, were characterized and tested as adsorbent forward to heavy and noble metals (12 elements). Different characterization techniques were employed.

### 3.1. Transmission Electron Microscopy (TEM)

**Figure 5:**
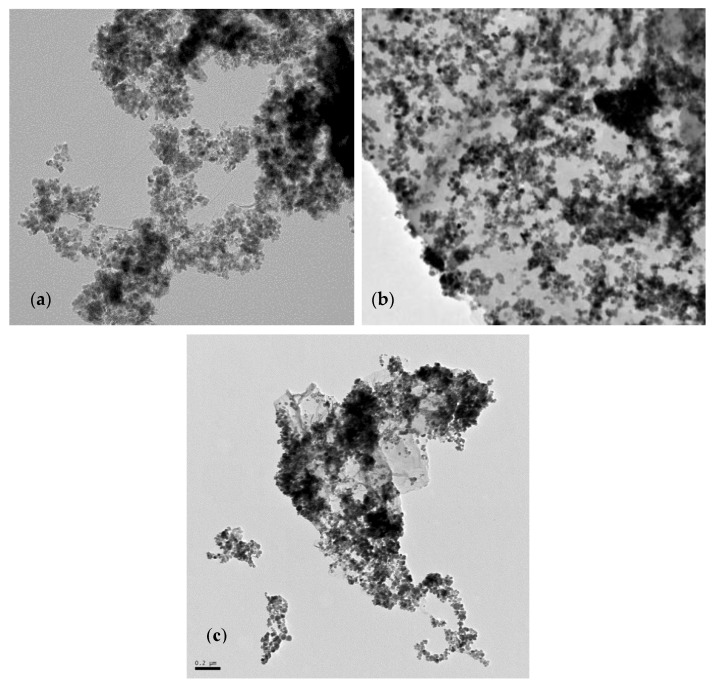
TEM images of materials synthesized by (**a**) Method 1 (1-PSTH), (**b**) Method 2 (2-PSTH) and (**c**) Method 3 (3-PSTH), with a scale of 0.2 μm.

The surface morphology of the synthesized materials was characterized by TEM and nitrogen adsorption isotherms. As an example, the TEM images of the materials functionalized with PSTH groups are shown in Figure 5; the TEM images of the DPTH materials were very similar. In these images, we can clearly observe that Fe_3_O_4_ nanoparticles were disorderly coupled over the GO sheets with a diameter between 12 and 20 nm. Nanoparticles’ size was selected intentionally, since smaller particles (<6 nm) show reduced saturation magnetization and magnetic susceptibility, due to surface effects that lower MRI relaxivity values, whereas larger particles (>20 nm) are difficult to disperse, owing to the presence of permanent magnetization at zero field [32]. The synthesis of adequate-sized MNPs was previously optimized by us [29].

### 3.2. N_2_ Adsorption/Desorption Isotherms

**Figure 6:**
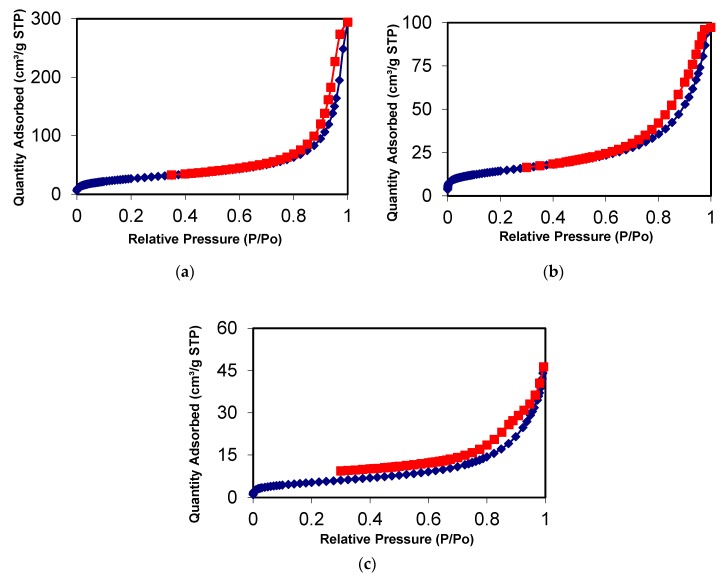
Nitrogen adsorption isotherm of materials synthesized by (**a**) Method 1 (1-PSTH), (**b**) Method 2 (2-PSTH) and (**c**) Method 3 (3-PSTH). Adsorption (blue line) and desorption (red line) processes.

The nitrogen adsorption-desorption experiments for all materials presented isotherms similar to those type IV, typical of mesoporous materials (pore size between 20 and 500 Å) [33]. The isotherms for the PSTH materials are shown in Figure 6. Some physics characteristics of the materials are shown in Table 1. As can be observed, the materials are mesoporous and present a surface area higher than non-coupled GO (2.63 m^2^/g). Method 3 materials presented lower surface areas than Methods 1 and 2 materials.

### 3.3. X-Ray Photoelectronic Spectroscopy (XPS)

**Figure 7:**
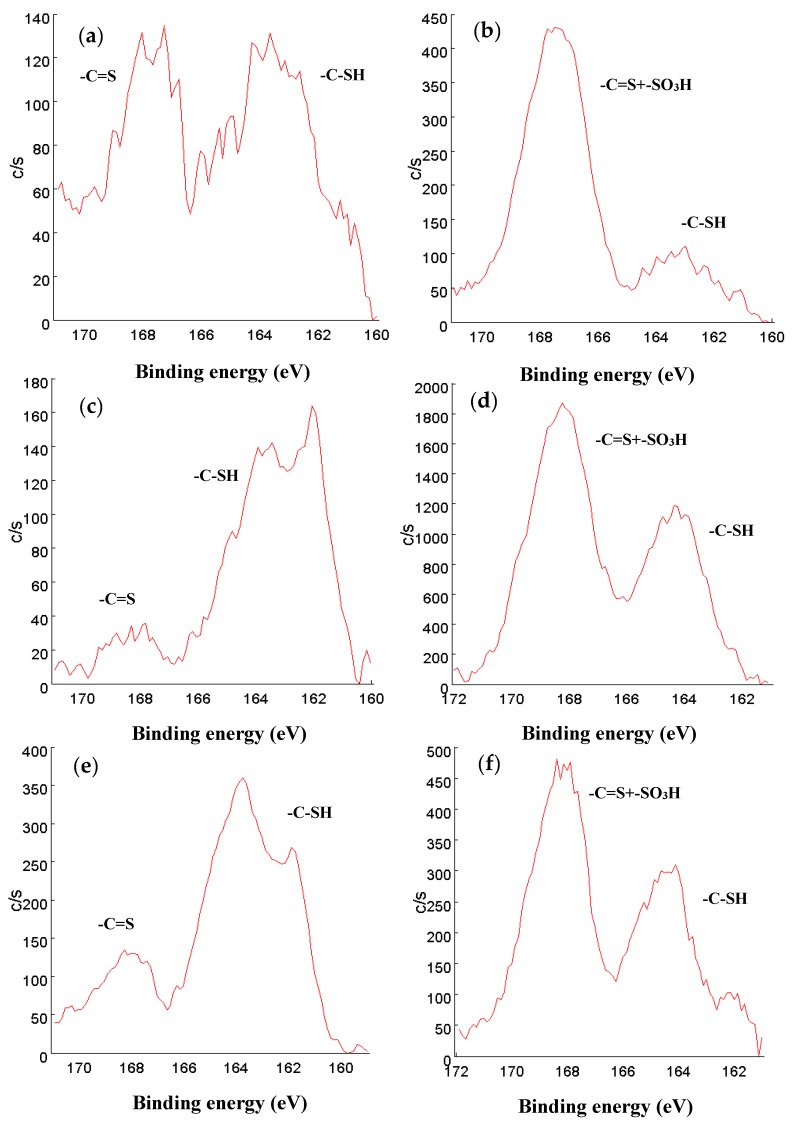
XPS sulfur spectra of (**a**) 1-DPTH; (**b**) 1-PSTH; (**c**) 2-DPTH; (**d**) 2-PSTH; (**e**) 3-DPTH; (**f**) 3-PSTH. Binding energy (eV) on *x* axis, and intensity of the signals (c/s) on *y* axis.

The XPS spectra in the sulfur region can be seen in Figure 7. In 1-DPTH, 2-DPTH and 3-DPTH spectra (Figure 7a,c,e, respectively), two oxidation states for sulfur are shown, being assigned to the tautomers of C=S (around 167 eV) and C-SH bond (around 163 eV). In 1-PSTH, 2-PSTH and 3-PSTH spectra (Figure 7b,d,f, respectively), the most energetic peak (more oxidized) is much higher due to the presence of a sulfonic group in the structure (Figure 4b,d). In the case of 2-DPTH, 2-PSTH, 3-DPTH and 3-PSTH (Figure 7c–f), the peak situated in a lower bind energy (C-SH bond) clearly presents a higher intensity in comparison with the equivalent peak in 1-DPTH and 1-PSTH spectra (Figure 7a,b). It can be concluded that the tautomeric equilibrium was displaced to the reduced tautomeric form (Figure 4c,d) when the materials were synthesized by Methods 2 and 3. The main explanation could be the presence of electronic interactions between the π–π system of GO and the aromatic system of the functional groups, favoring the tautomers with the most extended electronic system. This effect is stronger when the DPTH and PSTH are close to the GO sheet, meaning the functionalization through the –COOH groups of the GO sheet (Methods 2 and 3). In Method 1, the functional groups are attached only to the MNPs, resulting in a greater distance between the electronic systems.

**Figure 8:**
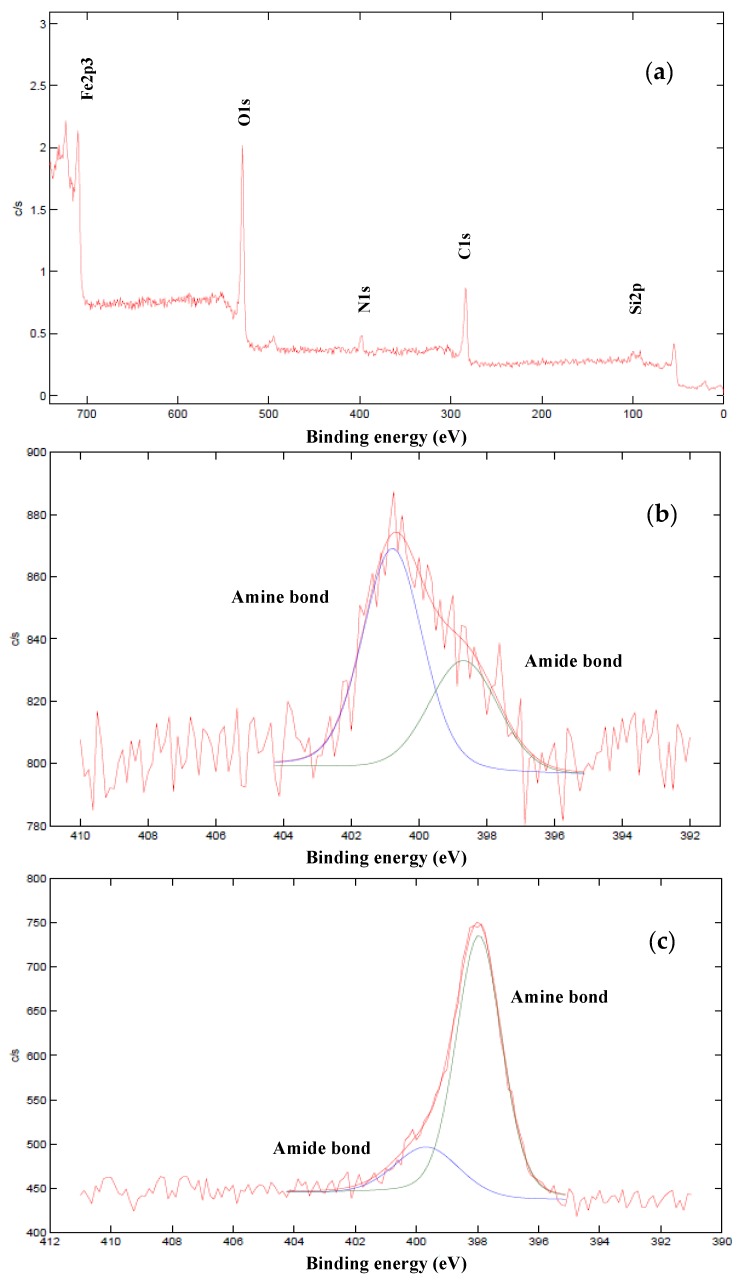
XPS spectra of Method 3: (**a**) EDA–MNPs–GO coupling; (**b**) deconvoluted XPS nitrogen spectrum of MNPs–GO coupling; and (**c**) deconvoluted XPS nitrogen spectrum of EDA–MNPs–GO coupling.

Figure 8 shows XPS spectra of Method 3: (a) EDA–MNPs–GO coupling; (b) deconvoluted XPS nitrogen spectrum of MNPs–GO coupling; and (c) deconvoluted XPS nitrogen spectrum of EDA–MNPs–GO coupling. The general views XPS spectra of all materials are similar to those shown in Figure 8a. These spectra confirm the double coupling of MNPs–GO in Method 3 (chemical and physical interactions). After the first step of the synthesis method (MNPs–GO coupling) two types of nitrogen are shown (Figure 8b), being assigned to amine groups (398.7 eV) and amide (400.8 eV) bonds [34]. Amide bonds resulted from the condensation reaction between aminopropyl groups of MNPs and –COOH functional groups on GO (chemical interaction) and the amine groups from the non-condensed aminopropyl groups of immobilized MNPs by physical interactions. In this way, the double coupling of MNPs–GO was demonstrated. As can be seen, the number of MNPs coupled by physical interactions was lower than MNPs coupled by chemical interactions (36% in front to 64% area, respectively). Figure 8c shows the sorbent after EDA–MNPs–GO coupling reaction and before functionalization of the material with the organic ligand. In this case, the peak assigned to amine bond is much higher than the peak assigned to amide bond (80% in front to 20% area, respectively), which confirms the presence of EDA in the structure.

### 3.4. CNHS-Fe Elemental Analysis

The MNPs–GO coupling and the functionalization grade of the new synthesized materials were studied by the determination of Fe concentration, using SS-HR CS GFAAS and CNHSO elemental analysis (Table 2). Method 3 showed a higher percentage of S and N, which confirms a better yield of functionalization than Methods 1 and 2. Moreover, 1-PSTH, 2-PSTH and 3-PSTH contain a higher percentage of S than 1-DPTH, 2-DPTH and 3-DPTH, respectively, due to the fact that PSTH presents an additional S atom in the structure (Figure 4b). Moreover, from the percentage of C, it could be concluded that Method 3 could bind a higher amount of GO to the material with 45% C, approximately; nevertheless, in Methods 1 and 2, the C percentages were lower (approximately, 8% and 30%, respectively). Furthermore, the Fe percentage was lower when C percentage increased in the structure, with both parameters being inversely proportional.

### 3.5. Mass Spectrometry

From MS spectra, the characteristic peaks were assigned to different fragments (Table 3). Some of them were found for every material, such as *m/z* = 129 and *m/z* = 207 peaks. Both were related to the presence of the thiocarbonohydrazide and pyridyl-thiocarbohydrazide, a reagent used in DPTH and PSTH synthesis. Other peaks were just found in the case of DPTH materials (*m/z* = 105 and 184) and PSTH materials (*m/z* = 64), agreeing with the structural differences between the functional groups.

### 3.6. Capacities of Adsorption of Heavy and Noble Metals from Aqueous Solution

In order to know which of these new nanomaterials offer better benefits to extract and preconcentrate metals ions, the capacities of adsorption toward 12 elements were studied (noble metals and transition metals). The capacities of adsorption of the materials were determined in batch, using the procedure described above (Section 2.6), by ICP-MS. From this study, it could be established that, at acid pH (Table 4), the highest values of adsorption were reached by 3-DPTH and 3-PSTH (Method 3) for each element. However, Method 3 materials presented lower surface areas than Method 1 and Method 2 materials (Table 1). This fact seems to explain that the main mechanism for the adsorption of metals is the chemisorption, not physisorption. Moreover, both materials reach the maximum value of the experiment (0.5 mg/g).

The capacities of adsorption of both materials were studied at higher concentration levels, and a second experiment was conducted (maximum level of 20 mg/g). In this case, the adsorption capacities of the materials were determined in batch by ICP-OES, and the data obtained are shown in Table 5. A comparison between non-functionalized GO and the materials synthesized by Method 3 was conducted to verify the effect of the functionalization and MNPs–GO coupling. As can be seen in Table 5, the prepared materials by Method 3 (3-DPTH and 3-PSTH) offered better benefits to be used as SPE adsorbent than non-functionalized GO at acid pH.

These benefits were obtained due to the double functionalization achieved in 3-DPTH and 3-PSTH synthesis; both MNPs and GO sheets were functionalized with the organic ligand. The ligands were anchored through the amine groups of: the aminopropyl silica coating of MNPs and the EDA–GO. This double functionalization was possible due to the double coupling. MNPs were coupled to the GO through two mechanisms: covalent binding by condensation with the carboxylic acid groups, and physical interactions with the GO adsorbent.

## 4. Conclusions

In this work, three different mechanisms for the coupling and functionalization of the MNPs and GO materials were addressed. For the synthesis of 1-DPTH and 1-PSTH, silica-covered MNPs were first functionalized, and after, they were dispersed on GO. For 2-DPTH and 2-PSTH synthesis, MNPs were coprecipitated into GO–EDA sheets before the functionalization of the GO layer. In 3-DPTH and 3-PSTH synthesis, both MNPs and GO sheets were functionalized with the organic ligand, and this process was called double functionalization. This double functionalization was possible due to the double coupling of MNPs and the GO layer (a fraction of MNPs coupled by Van der Waals interactions and a second fraction attached to the surface by covalent bond to the GO sheets), being the first time in which this double functionalization was addressed.

From the studies of the capacities of adsorption could be concluded that 3-DPTH and 3-PSTH showed higher capacity of adsorption toward different elements (transition and noble metals), being ideal adsorbents for decontamination purposes. Method 3 materials presented lower surface areas than Method 1 and Method 2 materials (Table 1). This fact seems to explain that the main mechanism for the adsorption of metals is the chemisorption, not physisorption. Therefore, the capacities of adsorption of the materials rely mostly on the superficial concentration of the functional groups. From the characterization studies was concluded that the functional groups were attached to the surface successfully, and the third procedure offered better yields of functionalization. Therefore, Method 3 was chosen as the more appropriate method to synthesize functionalized MNPs–GO coupling’s different functional groups, to improve the selectivity toward any chemical species.

## Figures and Tables

**Table 1:** Morphological information of the synthesized materials.

Material	1-DPTH	1-PSTH	2-DPTH	2-PSTH	3-DPTH	3-PSTH
Pore size (Å)	65.13	93.77	107.87	83.88	95.60	96.36
Surface area (m^2^/g)	64.30	99.17	46.94	52.14	37.88	19.58

**Table 2:** Elemental composition of the synthesized materials.

Sample	%C	%S	%N	%Fe
1-DPTH	8.681	0.121	0.657	51
1-PSTH	7.259	0.469	0.795	38
2-DPTH	27.046	1.087	4.638	46
2-PSTH	31.703	2.227	4.328	36
3-DPTH	41.658	3.604	8.205	31
3-PSTH	48.535	3.823	6.154	18

**Table 3:** MS spectra assignment of peaks.

Peaks (*m*/*z*)	Fragments
64	SO_2_^+^^∙^
105	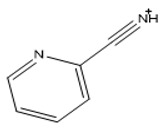
129	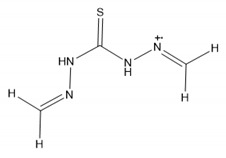
184	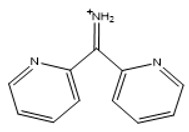
207	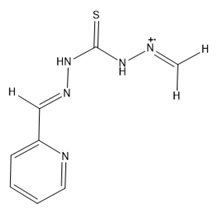

**Table 4:** Capacity of adsorption of the synthesized materials at acid pH (mg/g).

Element	1-DPTH	2-DPTH	3-DPTH	1-PSTH	2-PSTH	3-PSTH
Cr	0.0	0.1	0.5	0.0	0.2	0.5
Co	0.0	0.0	0.2	0.0	0.0	0.5
Ni	0.0	0.0	0.5	0.0	0.0	0.5
Cu	0.0	0.2	0.5	0.0	0.0	0.5
As	0.0	0.3	0.3	0.0	0.3	0.5
Cd	0.0	0.1	0.3	0.0	0.1	0.5
Hg	0.3	0.5	0.5	0.0	0.4	0.5
Pb	0.0	0.4	0.5	0.0	0.4	0.5
Pd	0.3	0.2	0.5	0.3	0.3	0.5
Ag	0.5	0.4	0.5	0.5	0.3	0.5
Pt	0.2	0.3	0.5	0.3	0.2	0.5
Au	0.5	0.3	0.5	0.4	0.2	0.5

The highest capacity of adsorption that could be obtained was 0.5 mg/g.

**Table 5:** Adsorption capacities of GO and materials synthesized by Method 3 at acid pH (mg/g).

Element	GO	3-DPTH	3-PSTH
Cr	0.3	0.5	0.5
Co	0.0	0.2	0.5
Ni	0.0	0.5	0.5
Cu	0.3	0.5	0.5
As	0.0	0.3	1.1
Cd	0.1	0.3	0.5
Hg	0.2	6.3	7.5
Pb	0.4	0.8	1.4
Pd	0.3	1.4	0.9
Ag	0.2	4.5	2.8
Pt	0.0	0.7	0.7
Au	0.0	8.7	12.8

The highest capacity of adsorption that could be obtained was 20 mg/g.
